# Sensory processing in autism: a call for research and action

**DOI:** 10.3389/fpsyt.2025.1584893

**Published:** 2025-05-21

**Authors:** Antonio Narzisi, Giacomo Vivanti, Stefano Berloffa, Annarita Milone, Pamela Fantozzi, Raffaella Tancredi, Gianluca Sesso, Valentina Viglione, Maddalena Fabbri Destro, Susanna Pelagatti, Luigi Olivero, Giulia Campatelli, Donata Pagetti, Gabriele Masi

**Affiliations:** ^1^ IRCCS Stella Maris Foundation, Calambrone, Pisa, Italy; ^2^ A.J. Drexel Autism Institute, Drexel University, Philadelphia, PA, United States; ^3^ IMT School for Advanced Studies, Lucca, Italy; ^4^ Consiglio Nazionale delle Ricerche, Istituto di Neuroscienze, Parma, Italy; ^5^ Department of Computer Science, University of Pisa, Pisa, Italy; ^6^ FISH-Toscana, Firenze, Italy

**Keywords:** autism spectrum disorder, sensory profile, hyper sensitivity, children, assessmennt

## 1 Introduction

On January 18, 2025, a significant multidisciplinary conference on Autism took place at the IRCCS Stella Maris Foundation in Pisa, Italy, a third-level university research hospital with nationwide catchment for child psychiatry. The conference, titled “Autism Spectrum Disorder and Neurodevelopment: Beyond Core Features – Second Edition”, attracted a diverse audience, including professionals from various fields, parents, individuals with autism, and trainees in child psychiatry and psychology. The event provided an essential platform for the exchange of knowledge and ideas, shedding light on various aspects of autism research and practice.

While this conference served as an important moment of reflection and exchange, it is not presented here as the scientific foundation of this manuscript. Rather, it acts as a contextual catalyst that draws attention to a topic already recognized as crucial within the scientific literature: sensory processing differences in autism.

A topic that emerged as particularly relevant during the final discussion was sensory processing, particularly oversensitivity. As the conversation unfolded among the speakers, the audience, and Temple Grandin— who was one of the keynote speakers —there was a collective recognition of the importance of this issue. Temple Grandin, who herself is on the autism spectrum and is also a prominent researcher and advocate in the field of autism, was asked by the audience what area of autism research she would prioritize for young researchers. Her response was unequivocal: “Research on treatments for heightened sensory sensitivity should be the top priority. Over sensitivity to stimuli, such as loud noises or scratchy clothes, causes pain and discomfort for many autistic individuals. There is a need for effective treatments to reduce over sensitivity.”

Grandin’s contributions to the understanding of sensory experiences in autism span both scientific research and personal narrative. Her work has provided valuable insights into the neurological basis of sensory processing differences and has informed intervention strategies, particularly through her writings and co-authored publications on sensory-based approaches (e.g., 1).

Her comment served as a compelling reminder of a widely acknowledged but still underprioritized research area. This opinion therefore builds upon existing empirical evidence and current clinical needs to advocate for a renewed and systematic scientific focus on sensory sensitivities in autism.

This statement by Grandin was not merely an insightful comment—it was a call to action. Sensory sensitivities affect the daily lives of individuals on the autism spectrum in profound ways. Despite its significance, research on effective interventions for sensory oversensitivity remains limited, and the issue is not given the attention it deserves. If we are serious about improving the quality of life for individuals with autism, it is essential to prioritize this area of research.

## 2 Why sensory processing should be a research priority?

Sensory processing differences are a core characteristic of autism. These differences influence how individuals with autism perceive and interact with their surroundings. For many, sensory oversensitivity presents as an aversion to loud noises, discomfort from bright lights, or even distress caused by specific textures, smells, or tastes. These heightened sensitivities can lead to severe distress, social withdrawal, and can significantly impair an individual’s ability to engage in daily activities or interact socially. As a result, sensory sensitivities often contribute to barriers to inclusion and full participation in society.

Despite the profound impact of sensory oversensitivity on the lives of autistic individuals, it remains an underexplored area in autism research. While sensory sensitivities are mentioned in diagnostic criteria for autism, the scientific community’s understanding of their underlying mechanisms is still in its early stages. Although sensory processing differences have long been observed in individuals with autism, and were eventually formalized in diagnostic frameworks such as the DSM-5 (2), the understanding of the mechanisms underlying these differences remains limited. Prior to DSM-5, sensory issues were not explicitly included in the diagnostic criteria despite being frequently reported in both clinical settings and research. Their formal inclusion reflects a growing awareness of their relevance but should not be mistaken for a complete understanding of the phenomenon. Why do some individuals with autism experience heightened sensitivity to sensory stimuli while others may be hyposensitive or exhibit fluctuating responses to sensory input? Are these sensitivities primarily the result of atypical neural processing, or do environmental and psychological factors play a significant role as well?

Current research has begun to suggest that sensory sensitivities may involve complex interactions between atypical sensory integration, increased neural connectivity, and dysregulation in sensory processing pathways in the brain (3). However, much remains unclear. Despite the growing recognition of sensory processing differences in autism, the lack of in-depth, targeted research leaves a gap in our understanding and limits the development of effective interventions.

This gap is especially evident when considering the relative scarcity of studies examining the longitudinal course of sensory sensitivities, their neurobiological correlates, and their response to targeted interventions. While early studies have described sensory symptoms in autism (e.g., 4–6), comprehensive, mechanistic research and large-scale clinical trials remain limited.

Furthermore, cultural and environmental factors often influence how sensory sensitivities are perceived and managed. For example, in environments with high sensory demands—such as noisy classrooms or busy workplaces—sensory sensitivities may be exacerbated. Conversely, sensory friendly settings that minimize environmental stimuli can significantly reduce distress and improve functioning for individuals with autism. Understanding how cultural, social, and environmental factors influence sensory experiences is crucial for creating interventions that are both effective and context-sensitive.

Addressing sensory sensitivities requires a coordinated approach across multiple levels of care. Primary care providers and pediatricians are often the first point of contact for families and can play a crucial role in early identification and referral. Specialized autism centers and neurodevelopmental clinics should be equipped to conduct comprehensive sensory assessments and deliver targeted interventions. Schools and educational settings must implement inclusive practices and provide sensory-friendly environments to support learning and social engagement. Families, who often serve as primary caregivers, need access to training and resources to manage sensory issues at home. Finally, community services, including mental health and occupational therapy providers, can offer support through structured programs and outreach. A multi-tiered, multidisciplinary approach is essential to ensure that interventions are accessible, sustainable, and tailored to individual needs.

## 3 Moving beyond recognition: the need for action

Despite the growing recognition of sensory processing differences, the research community has yet to prioritize the development of effective solutions. Current interventions, such as sensory integration therapy and environmental modifications, have shown promise in improving sensory regulation for some individuals with autism. However, these interventions often lack sufficient empirical support, leaving practitioners and families with limited guidance on how to best manage sensory sensitivities.

It is important to clarify that this opinion does not present original empirical findings, but instead advocates for a targeted research agenda. The discussion around novel stimulation techniques and sensory enrichment is therefore intended as a forward-looking recommendation based on preliminary evidence and an urgent clinical need. Sensory processing challenges are known to persist or fluctuate across the lifespan in many individuals with autism. This ongoing vulnerability further justifies the development of innovative, empirically supported strategies such as sensory-enriched environments and adaptive technologies.

To address this gap, there is a need for a concerted effort to advance research in this area. Several critical actions are needed to guide future investigation and inform clinical practice.

Existing studies already provide a valuable foundation for the proposed research directions. For example, Green et al. (3) identified over-reactive responses in the amygdala and somatosensory cortex in autistic youth exposed to sensory stimuli, highlighting altered sensory processing at the neural level. Similarly, Woo and Leon (7) found that environmental enrichment led to measurable improvements in sensory responsiveness in children with autism. These results underscore both the presence of neurobiological differences and the potential for targeted interventions, while also revealing gaps that require larger-scale, longitudinal, and context-sensitive studies to inform clinical and policy decision-making.

Specifically, researchers, clinicians, and policymakers should focus on the following priorities:

(a) *Investigating underlying neural mechanisms.* While we have some insights into the neural bases of sensory sensitivities, further studies are needed to unravel the specific brain mechanisms at play.

Neuroimaging studies, for example, could help identify brain activity patterns that correspond to heightened sensory responses, providing crucial insights into the neurological underpinnings of these sensitivities. For instance, Green et al. (3) found that youth with autism showed over-reactive responses in brain regions such as the amygdala and somatosensory cortex when exposed to sensory stimuli. These findings support the hypothesis of altered sensory processing pathways and highlight the potential of neuroimaging to identify neural correlates of sensory sensitivity in autism.

(b) Developing evidence-based interventions. Interventions aimed at managing sensory oversensitivity must be scientifically validated. This includes rigorously testing sensory-friendly environments and wearable adaptive technologies tailored to each person’s unique sensory profile.

These interventions should be tested in diverse real-world settings, including homes, schools, and workplaces. Recent studies suggest that sensory-based interventions may help modulate sensory reactivity and support functional outcomes in children with autism (7, 8), although further validation through controlled, longitudinal studies is still needed.

(c) Engaging individuals with autism in research. To ensure that research efforts are truly addressing the needs of individuals with autism, it is essential to involve people with autism and their families directly in the research process. This means conducting qualitative studies that capture lived experiences and using these insights to shape the design and implementation of interventions.

(d) Exploring longitudinal effects. Sensory sensitivities may evolve as individuals with autism grow older. To effectively address these challenges, a diverse range of study design is needed. While some studies, such as Tavassoli et al. (9), have begun to explore changes in sensory symptoms over time, the number of robust longitudinal investigations remains limited. Recent studies by Dellapiazza et al. (10) and Lau et al. (11) provide important new data on the developmental course of sensory processing differences, highlighting their variability and potential persistence across childhood. In contrast, studies such as Baranek et al. (12) offer valuable cross-sectional insights by comparing sensory responsiveness among autistic children, children with developmental delays, and neurotypical peers. These findings, while not longitudinal in design, nonetheless contribute to the understanding of early sensory phenotypes.

A comprehensive approach to future research should integrate longitudinal, cross-sectional, and qualitative methodologies to fully capture the complexity and individual variability of sensory processing across the lifespan.

These studies can provide insights into developmental trajectories and inform timing and targets for interventions. Randomized controlled trials (RCTs) are essential to rigorously assess the efficacy of both behavioral and environmental interventions, such as sensory integration therapy or the use of adaptive technologies. Neuroimaging studies, including fMRI and EEG, can help identify neural markers associated with different sensory profiles, contributing to a neurobiological understanding of these phenomena. Additionally, qualitative studies involving interviews with autistic individuals and their families can offer rich, first-hand perspectives that inform the design and implementation of interventions, ensuring that research remains grounded in real-world experiences.

In addition to these research priorities, it is also important to examine the role of environmental enrichment and therapy in mitigating sensory sensitivities. Studies such as the randomized controlled trial by Woo and Leon (7) demonstrate that environmental enrichment can help manage sensory sensitivities, while Ayres Sensory Integration Therapy has shown promise in several clinical contexts (8, 9, 13). However, there remains a need for more robust studies that validate the efficacy of such interventions.

## 4 Conclusion: a research imperative

Temple Grandin’s call for prioritizing research into sensory sensitivity is not just a suggestion; it is an urgent necessity. Sensory sensitivities significantly impact many aspects of life for individuals with autism, including education, social inclusion, employment, and mental health. Yet, despite their profound impact, they remain a largely neglected area of research.

If we are genuinely committed to improving the lives of individuals with autism, we must go beyond merely acknowledging the issue of sensory sensitivities. We need to invest in research that will lead to the development of effective, evidence-based interventions. These interventions should be designed not only to alleviate distress but also to empower individuals with autism to better navigate and engage with the world around them. The time for action is now. Only through rigorous scientific inquiry and collaboration can we hope to develop the tools necessary to enhance the lives of individuals on the autism spectrum and ensure their inclusion and well-being.
